# The effectiveness of electrical stimulation for the management of benign prostatic hyperplasia: A protocol for systematic review and meta analysis

**DOI:** 10.1097/MD.0000000000019921

**Published:** 2020-05-08

**Authors:** Wei-jun Han, Yu-ge Guo, Yun-qi Wang, Jin-wan Wang

**Affiliations:** a Third Ward of Surgery Department, Baoji Hospital of Traditional Chinese Medicine, Baoji; b Department of Obstetrics and Gynecology; c Department of Urology, Yangling Demonstration District Hospital, Xianyang, China.

**Keywords:** benign prostatic hyperplasia, effectiveness, electrical stimulation, randomized controlled trial, safety

## Abstract

**Background:**

This study will aim to assess the effectiveness and safety of electrical stimulation (ES) for the treatment of patients with benign prostatic hyperplasia (BPH).

**Methods:**

PubMed, EMBASE, Web of science, Springer, Cochrane Library, PsycINFO, Allied and Complementary Medicine Database, CBM, and China National Knowledge Infrastructure will be retrieved from inception to the September 1, 2019. No language limitation will be applied to this study. Randomized controlled trials (RCTs) that assessed the effectiveness and safety of ES for the treatment of patients with BPH will be considered for inclusion. Literature selection, data collection, and risk of bias assessment will be conducted by 2 investigators independently. Statistical analysis will be carried out using RevMan 5.3 Software.

**Results:**

This study will summarize high quality RCTs based on the present evidence of ES for the treatment of BPH in several aspects, including changes in urological symptoms, changes in prostate size, urodynamic parameters, quality of life, and number and severity of adverse events.

**Conclusion:**

The findings of this study will provide latest evidence to appraise whether ES is an effective and safety intervention for patients with BPH.

**PROSPERO registration number:**

PROSPERO CRD42019157241.

## 1 Introduction

Benign prostatic hyperplasia (BPH) is one of the most common disorders among male population.^[1–4]^ Although it is not a life-threatening condition, it often leads to serious morbidity in the male elderly.^[5–7]^ It has been estimated that >50% of them suffer from this disorder.^[8]^ Several risk factors are responsible for such condition, including aging, family history, diabetes and heart disease, and lifestyle.^[9–15]^

A numerous studies have reported that electrical stimulation (ES) can be utilized for the treatment of many diseases, such as spinal cord injury, pressure ulcers, stroke, aphasia, obstructive sleep apnoea, overactive bladder, and BPH.^[16–24]^ Previous clinical trials have found that ES can benefit patients with BPH.^[24–27]^ However, their results are still inconsistent. Thus, this study will systematically assess the effectiveness and safety of ES for the treatment of BPH.

## 2 Methods and analysis

### 2.1 Study registration

We have registered this study on PROSPERO (CRD42019157241). It has been reported follows the guidelines of Preferred Reporting Items for Systematic Review and Meta-Analysis Protocols Statement.^[28]^

### 2.2 Study inclusion and exclusion criteria

#### 2.2.1 Types of studies

All relevant randomized controlled trials (RCTs) on assessing the effectiveness and safety of ES for the treatment of BPH will be included without language restriction. We will not consider studies of non-clinical studies, non-controlled trials, non-RCTs, and quasi-RCTs.

#### 2.2.2 Types of interventions

The patients in the experimental group received any forms of ES, regardless dosage, frequency, and duration.

The patients in the control group received any interventions, except any forms of ES.

#### 2.2.3 Types of participants

Patients who have diagnosed with BPH will be included with no limitations of country, race, and age.

#### 2.2.4 Types of outcome measurements

Primary outcome is change in urological symptoms, as assessed using International Prostate Symptom Score or other relevant scales. Secondary outcomes are changes in prostate size; urodynamic parameters (including residual urine volume (mL), mean urine flow [mL/s]); quality of life, as measured by any relevant tools; and number and severity of adverse events.

### 2.3 Search methods for the identification of studies

#### 2.3.1 Electronic databases

The databases of PubMed, EMBASE, Web of science, Springer, Cochrane Library, PsycINFO, Allied and Complementary Medicine Database, CBM, and China National Knowledge Infrastructure will be retrieved from inception to the September 1, 2019. All of those electronic databases will be searched without restrictions of language and publication status. The search terms include benign prostatic hyperplasia, lower urinary tract symptoms, prostate gland enlargement, urinary symptoms, electrical stimulation, functional electrical stimulation, electromyostimulation, electrical muscle stimulation, neuromuscular electrical stimulation, randomized controlled trial, random, randomly, blind, concealment, and controlled study. The retrieval detailed strategy for PubMed is showed in Table 1. Identical search strategy for other electronic databases will also be adapted and built.

**Table 1:**
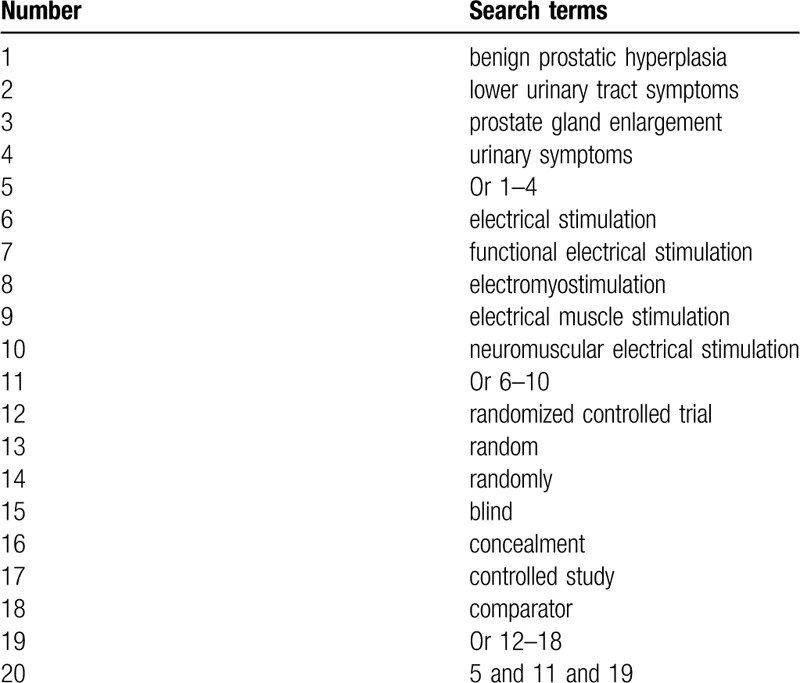
Search strategy sample of PubMed.

#### 2.3.2 Other literature sources

We will also search dissertations, ongoing studies, reference lists of relevant reviews, and included studies.

### 2.4 Data collection and analysis

#### 2.4.1 Selection of studies

**Figure 1:**
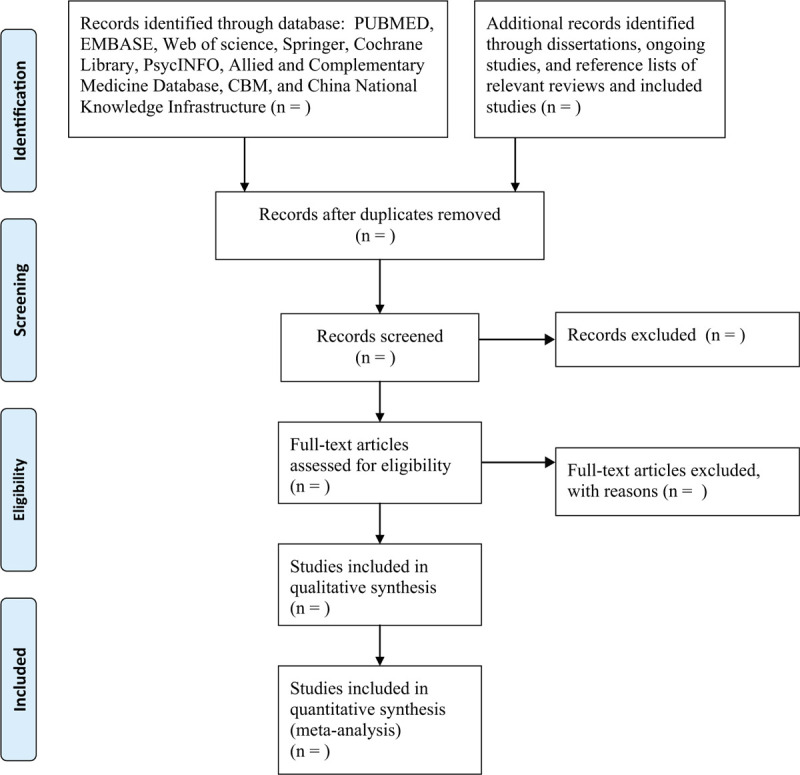
Flowchart of study selection.

All searched records will be imported into the Endnote X8 and all duplicated studies will be excluded. Two investigators will independently screen titles and abstracts based on the previously designed eligibility criteria, and irrelevant studies will be removed. The remaining studies will be read by full texts to further judge if they meet all inclusion criteria. Studies with incomplete, or insufficient or unclear or missing information will be contacted original corresponding authors to obtain them. Any divergences will be solved with the help of a third investigator when necessary. The results of study selection will be presented in Fig. 1.

#### 2.4.2 Data extraction and management

Two investigators will independently extract data using standard data extraction sheet. The extracted data consists of study information (first author, year of publication, journal, location, etc), patient characteristics (age, sex, diagnostic criteria, inclusion and exclusion criteria, etc), study methods (randomization, blind, allocation, etc), intervention details (types of treatments, dosage, frequency, etc), all outcome measurements, and safety. In case of any divergences, a third investigator will be consulted for assistance.

### 2.5 Risk of bias assessment

Two investigators will assess the risk of bias for each study using Cochrane risk of bias tool. It has 7 items, and each item is further divided into low risk of bias, unclear risk of bias, and high risk of bias. If there are any different opinions, we will invite a third investigator to solve them by discussion.

### 2.6 Statistical analysis

We will use RevMan 5.3 software, Cochrane Community, London, UK to analyze extracted data. Mean difference and standard mean difference and 95% confidence intervals (CIs) will be utilized to estimate continuous data. Risk ratio and 95% CIs will be used to calculate dichotomous data. We will assess heterogeneity among eligible studies using *I*^2^ statistic. *I*^2^ ≤ 50% is considered as representing reasonable heterogeneity, and a fixed effect model is used for analysis. *I*^2^ > 50% is regarded as having significant heterogeneity, and a random-effects model is utilized for statistics. We will perform a meta-analysis if heterogeneity is reasonable among sufficient eligible RCTs (often >2 studies) with identical characteristics, interventions, controls, and outcomes are included. If there is substantial heterogeneity, we will conduct a subgroup analysis and a meta-regression test. If significant heterogeneity still exists after subgroup analysis, we will not conduct a meta-analysis. Instead, we will report outcome results as a narrative summary based on the Guidance on the Conduct of Narrative Synthesis in Systematic Reviews.

### 2.7 Subgroup analysis

We will carry out a subgroup analysis to identify any possible causes for the substantial heterogeneity based on different types of interventions, controls, and outcome measurements.

### 2.8 Sensitivity analysis

We will take a sensitivity analysis to test the robustness and stability of pooled study results by excluding low quality studies.

### 2.9 Reporting bias

When there are sufficient eligible RCTs (≥10 studies), we will carry out funnel plot and Egger regression test to test reporting bias.^[29,30]^

### 2.10 Ethics and dissemination

It is not necessary to provide ethical approval for this study, because all data of this study will be extracted from previous published records. We will publish this study at a peer-reviewed journal.

## 3 Discussion

ES therapy has several characteristics of few side effects, inexpensiveness, and easy acceptability. It can effective relieve symptoms for patients with BPH. A variety of studies have reported that ES is used for the BPH treatment very efficacious.^[24–27]^ However, its conclusion is still inconsistent, and no systematic review has been addressed to explore this issue. Therefore, this study will systematically evaluate the effectiveness and safety of ES for the treatment of patients with BPH. It will summarize recent high quality evidence to judge whether ES is effective and safety for BPH. Its results may provide helpful evidence for both clinician and patients.

## Author contributions

**Conceptualization:** Wei-jun Han, Yu-ge Guo, Yun-qi Wang.

**Data curation:** Wei-jun Han, Yun-qi Wang, Jin-wan Wang.

**Formal analysis:** Wei-jun Han, Yu-ge Guo.

**Investigation:** Yu-ge Guo, Yun-qi Wang.

**Methodology:** Wei-jun Han, Yu-ge Guo.

**Project administration:** Yun-qi Wang.

**Resources:** Wei-jun Han, Yu-ge Guo, Jin-wan Wang.

**Software:** Yu-ge Guo, Jin-wan Wang.

**Supervision:** Yun-qi Wang.

**Validation:** Wei-jun Han, Yu-ge Guo, Yun-qi Wang, Jin-wan Wang.

**Visualization:** Wei-jun Han, Yun-qi Wang.

**Writing – original draft:** Wei-jun Han, Yu-ge Guo, Yun-qi Wang, Jin-wan Wang.

**Writing – review & editing:** Wei-jun Han, Yu-ge Guo, Yun-qi Wang.
